# More Delusions May Be Observed in Low-Proficient Multilingual Alzheimer’s Disease Patients

**DOI:** 10.1371/journal.pone.0140714

**Published:** 2015-11-10

**Authors:** Yi-Chien Liu, Yen-Ying Liu, Ping-Keung Yip, Kyoko Akanuma, Kenichi Meguro

**Affiliations:** 1 Division of Geriatric Behavioral Neurology, CYRIC, Tohoku University, Sendai, Japan; 2 Fu Jen University School of Medicine, Taipei, Taiwan; 3 Neurological Center of Cardinal Tien Hospital, Taipei, Taiwan; University of Electronic Science and Technology of China, CHINA

## Abstract

**Background:**

Language impairment and behavioral symptoms are both common phenomena in dementia patients. In this study, we investigated the behavioral symptoms in dementia patients with different language backgrounds. Through this, we aimed to propose a possible connection between language and delusion.

**Methods:**

We recruited 21 patients with Alzheimer’s disease (AD), according to the DSM-IV and NINCDS-ADRDA criteria, from the memory clinic of the Cardinal Tien Hospital in Taipei, Taiwan. They were classified into two groups: 11 multilinguals who could speak Japanese, Taiwanese and Mandarin Chinese, and 10 bilinguals who only spoke Taiwanese and Mandarin Chinese. There were no differences between age, education, disease duration, disease severity, environment and medical care between these two groups. Comprehensive neuropsychological examinations, including Clinical Dementia Rating (CDR), Mini-Mental Status Examination (MMSE), Cognitive Abilities Screening Instrument (CASI), Verbal fluency, Chinese version of the Boston naming test (BNT) and the Behavioral Pathology in Alzheimer’s Disease Rating Scale (BEHAVE-AD), were administered.

**Results:**

The multilingual group showed worse results on the Boston naming test. Other neuropsychological tests, including the MMSE, CASI and Verbal fluency, were not significantly different. More delusions were noted in the multilingual group. Three pairs of subjects were identified for further examination of their differences. These three cases presented the typical scenario of how language misunderstanding may cause delusions in multilingual dementia patients. Consequently, more emotion and distorted ideas may be induced in the multilinguals compared with the MMSE-matched controls.

**Conclusion:**

Inappropriate mixing of language or conflict between cognition and emotion may cause more delusions in these multilingual patients. This reminds us that delusion is not a pure biological outcome of brain degeneration. Although the cognitive performance was not significantly different between our groups, language may still affect their delusion.

## Background

Among the symptoms of Alzheimer’s disease (AD), symptoms of psychosis, such as delusion, are most disturbing and stressful for caregivers. According a previous study, 30–40% of patients suffer from some degree of behavioral symptoms during the stage of mild cognitive impairment (MCI) or mild dementia [1]. Psychotic symptoms in early stage AD patients usually means a worse outcome and early institutionalization [2]. Specific type of delusion content is related to dysfunction of different brain areas [3]. Some types of delusion, such as “residence is not home”, are specifically related to cognitive decline [4]. Post-mortem pathology has shown that AD patients with delusion exhibit more plaques and tangles in the frontal lobe [5].

In addition to behavioral symptoms, language impairment is also a common and well-known symptom in MCI or mild AD [6]. Previous reports have also demonstrated that cognitive impairment, such as language, may have predictive value for behavioral symptoms [7]. Moreover, the effect of language impairment is not symmetric across every language [8]. A study of Japanese-Portuguese bilingual patients also reflected different patterns of degeneration in each of their languages. During the progression of AD, the Japanese kanji writing system was affected more than Portuguese [9]. Another study performed in Spanish-English bilinguals also demonstrated different degenerated pattern between dominant and non-dominant language [10]. This kind of asymmetric degeneration may cause even more problems for multilingual AD patients.

The impact of language impairment in AD patients is not only restricted to communication but is also related to behavioral problems, such as delusion [11]. During the progression of AD, the neuropsychological findings of bilinguals are not different from monolinguals [12]. However, it remains unknown whether the clinical course or behavioral symptoms will differ between bilinguals and monolinguals. In addition to its role in communication, language can also play a constructive role in emotion perception [13]. Furthermore, more and more new findings suggest that emotion and motivation may significantly influence both delusion and confabulation [14].

The aim of our study was to attempt to understand if language has an impact on behavioral symptoms in multilingual AD patients; focusing particularly on people who are not fully multilingual, which means their language fluency remains at a low level.

Before WWII, many Taiwanese received formal Japanese education. Thus, they can speak Japanese, compared with their Taiwanese peers who may receive education after the war or in Mainland China. After the war, the official language of Taiwan changed to Mandarin Chinese. As a result, their Japanese remained at a low fluency level [15]. This study provides us with the opportunity to investigate whether these early language exposures or experience could exert any influence on dementia symptoms, especially behavioral symptoms.

## Methods

### Participants

**Fig 1:**
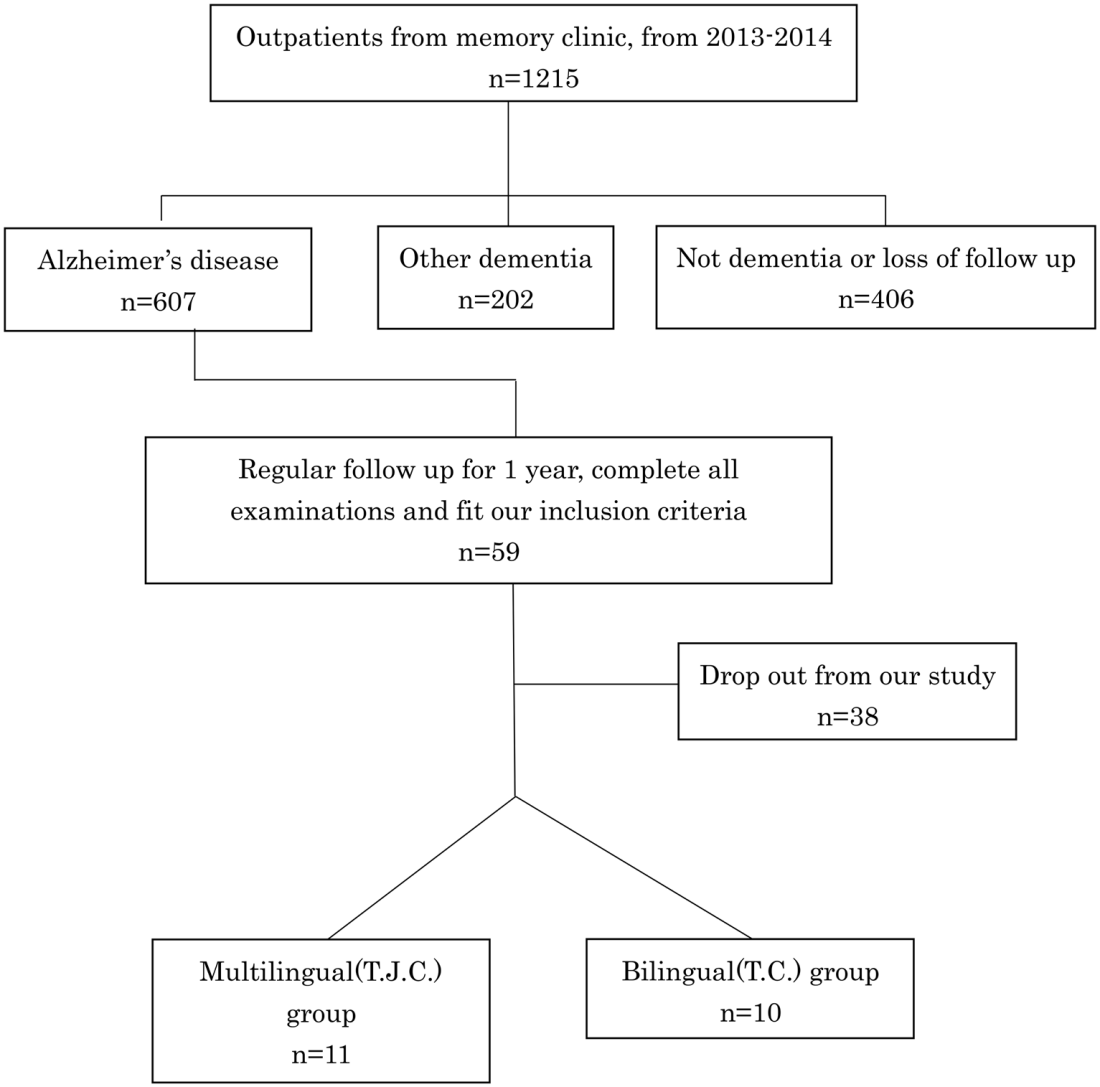
Flow chart of the subjects’ selection from the memory clinic. From 2013–2014, 59 dementia patients from the memory clinic that met our inclusion criteria were selected. Ultimately, 21 patients agreed to participate in our study.

From 2013–2014, 59 subjects who underwent regular follow up and met the following inclusion criteria were recruited from the memory clinic of Cardinal Tien hospital in Taipei, Taiwan. Ultimately, 21 subjects joined our study and provided signed informed consent. All of our subjects were cared for by their family member and none of them had ever lived in nursing home. They all lived in nearby area in Taipei and their caregiver was generally their son or daughter. They were selected through the following flow chart (Fig 1).

### Definition of multilingualism

Multilingualism was defined as the ability to fluently use Taiwanese (T), Japanese (J) and Mandarin Chinese (C) in their daily life. Before dementia, they had no problems in communicating with other people and understand their meanings in each of these languages. Most of our multilingual (T.J.C.) subjects received formal education of Japanese before WWII. After the war, some of them were educated in Mandarin Chinese, when the official language changed to Mandarin Chinese; therefore, most of them learned to use it in everyday life. However, Japanese was the first symbolized language they learned. Thus, they continued to read the newspaper and write post cards to their friends in Japanese; these facts were confirmed by their family. The Japanese level of the multilingual (T.J.C.) subjects was evaluated by a Japanese teacher. Their Japanese level was generally 2–3/5 (0 to none and 5 to perfect). In another words, their Japanese level was of low fluency. In contrast, their age-matched bilingual (T.C.) controls were only fluent in Taiwanese and Chinese. They had not received any prior Japanese education. Most of them depended on Mandarin Chinese for their everyday life, including business, government information or letter writing. This was also confirmed by their family members.

### Inclusion criteria

1. All of the patients were diagnosed with probable Alzheimer’s disease of mild severity, according to DSM-IV and NINCDS-ADRDA criteria.
2. The disease duration was two years for all of the subjects and all of them had to be followed up regularly in the memory clinic for at least one year in order to confirm the diagnosis.

### Exclusion criteria

1. Routine medical checkups, including 1.5-Tesla magnetic resonance imaging (MRI), electrocardiogram, chest X-ray and laboratory tests of urine and blood were arranged for all subjects. If any possible reversible cause of dementia was found, they were excluded from the study.
2. Patients with a history of psychiatric disease, such as schizophrenia.
3. Patients who were being treated with any kind of typical or atypical antipsychotic medication.

### Ethics

The individual in this manuscript has given written informed consent (as outlined in PLOS consent form) to publish these case details. The study was approved by the Ethics Committees of Tohoku University, Sendai, Japan and Cardinal Tien hospital, Taipei, Taiwan. Signed informed consent was provided by the patients themselves or their families.

### Neuropsychological assessment

#### General cognitive function evaluation

All patients underwent comprehensive neuropsychological screening, including the Clinical dementia rating scale (CDR), the Mini-Mental Status Examination (MMSE), and the Cognitive Abilities Screening Instrument (CASI). All of above tests were performed during the first visit to the memory clinic.

#### Language function evaluation

Because most of our subjects were 80 years of age or more, they could not maintain their concentration for too long. Thus, we chose the Chinese version of the Boston naming test, which has been validated for users among the Chinese speaking population [16]. For the Boston naming test, subjects could respond to the picture presented to him/her in Japanese/Taiwanese/Mandarin Chinese, and their responses were recorded. Verbal fluency tests were performed with 3 categories (fruit, vegetable and fish), and our subjects were asked to respond to as many as possible within one minute (in each category). The Boston naming test was performed after confirmation of the diagnosis of each of the subjects in our clinic.

### Behavioral symptoms evaluation

In the assessment of the behavioral symptoms, the standard Chinese version of the Behavioral Pathology in Alzheimer’s disease rating scale (BEHAVE-AD) was used [17]. The component of delusion was also recorded as the following categories: delusion of theft, "this is not my home" delusion, delusion of infidelity, misidentification syndrome (Capgras syndrome), suspiciousness and others. The BEHAVE-AD test was also administered after the diagnosis of our subjects was confirmed in our clinic.

### MRI assessment

Each participant underwent brain MRI (GE Signa EXCITE 1.5T MRI, GE Taiwan) in order to exclude organic lesion and to evaluate the extent of their medial temporal lobe atrophy. This evaluation was made using a visual rating scale [18].

### Statistical analysis

Statistical analysis was conducted using IBM SPSS Statistics 22 to examine the relationships demographic and neuropsychological data between multilingual and bilingual groups. Due to small number of subjects in this study, non-parametric statistics (Mann-Whitney U-test) were used to detect significant difference. All the data used in these analyses is available in the supporting information (S1 File).

## Results

### Demographic data and neuropsychological findings

The age, education, gender and dementia severity did not differ between groups. The results of the Boston naming test were better in the control group, with statistically significant differences. The other neuropsychological tests, including MMSE, CASI and Verbal fluency, showed no differences between groups (see Table 1). Behavioral symptoms were noted more often in multilingual patients compared with controls. However, the most significant difference was noted in delusion (see Table 1).

**Table 1:** Demographic and neuropsychological test results.

	Multilingual (T.J.C.)	Bilingual (T.C.)	P value
Number	11	10	
Age	83.7 (2.9)	81.5 (7.9)	0.3
Gender (% women)	63%	50%	
Education	9.7 (3.2)	10.8 (5.1)	0.7
MMSE^2^	19.5 (3.3)	20.3 (3.2)	0.4
CASI^3^	60.1 (11.4)	66.5 (12.8)	0.3
Memory domain of CASI	4.3(2.2)	5.2(1.7)	0.3
Verbal fluency^4^	6.4 (2.3)	6.9 (1.8)	0.5
Boston naming test (spontaneous)	38.8 (8.0)	45.8 (6.4)	0.09
Boston naming test (total)	43.6 (9.1)	51.4 (6.4)	0.03
BEHAVE-AD^5^ scores	12.8 (8.1)	1.9 (3.2)	0.001
Delusion^6^	4.18 (2.72)	0.3 (0.94)	<0.001

**Note:** For both group of our subjects, their age, education, MMSE, CASI, Verbal fluency, BNT results, the mean (SD) is presented. The abbreviation is as following.

^1^ T.J.C.: Taiwanese, Japanese, Mandarin Chinese, T.C.: Taiwanese and Mandarin Chinese

^2^ MMSE: Mini-mental status examination

^3^ CASI: Cognitive abilities screening instrument

^4^ Verbal fluency: Names of 3 category of vegetable, fruit and fish are asked to answer in 1 minute, the score here is the mean

^5^ BEHAVE-AD: Behavioral pathology in Alzheimer’s disease

^6^ Delusion: Sub-item scores in BEHAVE-AD.

### Case description

**Fig 2:**
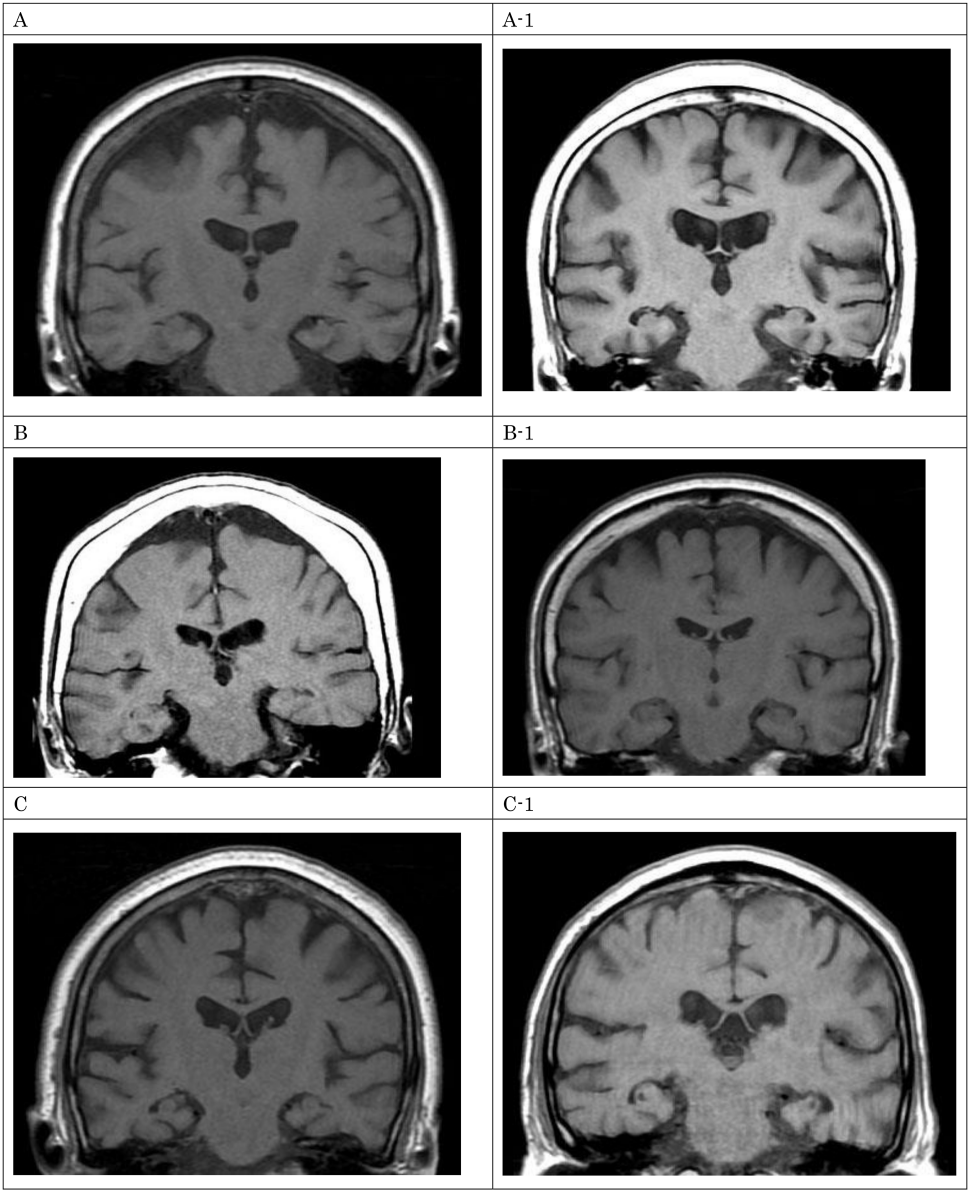
MRI of multilingual cases (A, B, C) and their controls (A-1, B-1, C-1), the degree of medial temporal lobe atrophy did not differ between the groups. The coronal views (perpendicular to the line intersecting the anterior and posterior commissures in the midsagittal plane) of the T1-weighted MRI image are illustrated in each group.

To better illustrate our study findings, we matched 3 multilingual (T.J.C.) AD patients with bilingual (T.C.) controls, based upon their MMSE scores (see Table 2). The content of delusion in three multilingual patients was also described in detail, along with the neuroimaging findings of the medial temporal lobe (see Fig 2).

**Table 2:** Demographic characteristics for MMSE-matched pairs of the multilingual and bilingual AD patients.

	MMSE 22 and 24		MMSE 18 and 20		MMSE 14 and 16	
Case	A	A-1	B	B-1	C	C-1
Age	80	81	80	71	83	83
Education	9	9	12	4	6	6
MMSE	22	24	20	18	14	16
CASI	75	68	60	59	50	57
Boston naming	39	56	46	51	40	41
MTA score	1	2	1	1	2	2
BEHAVE-AD	19	0	9	0	26	0
Delusion	7	0	1	0	8	0

**Note**: Three pairs of multilingual (T.J.C.) subjects (A, B, C) and their MMSE-matched bilingual (T.C.) controls (A-1, B-1, C-1) were illustrated for better demonstration. In each pair, their neuropsychological test didn’t differ from each other. But multilingual subjects (A, B, C) had more behavioral symptoms, especially delusion. The abbreviation of MTA score means score of visual rating scale of medial temporal lobe.

### Case A

#### Summary of the clinical history and related language background

Age: 80

Chief complaint: Poor memory and persistent delusion

Past medical history: Idiopathic hypertension under regular control

Current diagnosis: amnesic MCI

Disease duration: 2 years

Previous Job before retirement: Nurse

**Fig 3:**
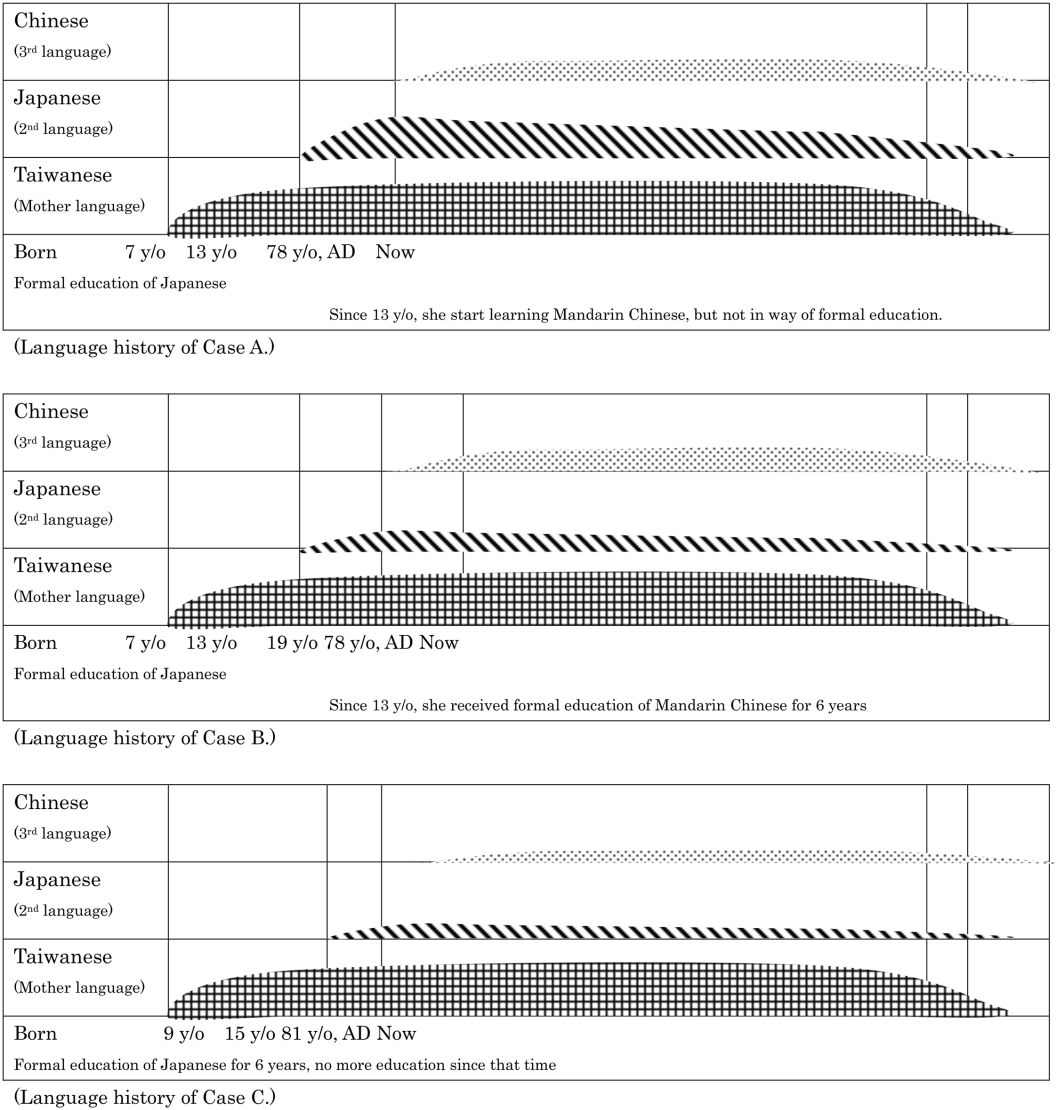
Asymmetric language degeneration in our multilingual subjects. Multilingual subjects received formal education in Japanese for at least 6 years and Mandarin Chinese with variable courses. During the progression of dementia, impaired language ability was apparent in each language. However, their language ability was not equal at the outset. The result is a very asymmetric language presentation after dementia.

Language use in daily life: Mostly she communicated with people in Taiwanese. But she talked with her husband and their friends in Japanese. She could only read and write in Japanese. She rarely used Mandarin Chinese, especially after she retired. (See Fig 3)

#### Detailed content of delusion in Case A

**Fig 4:**
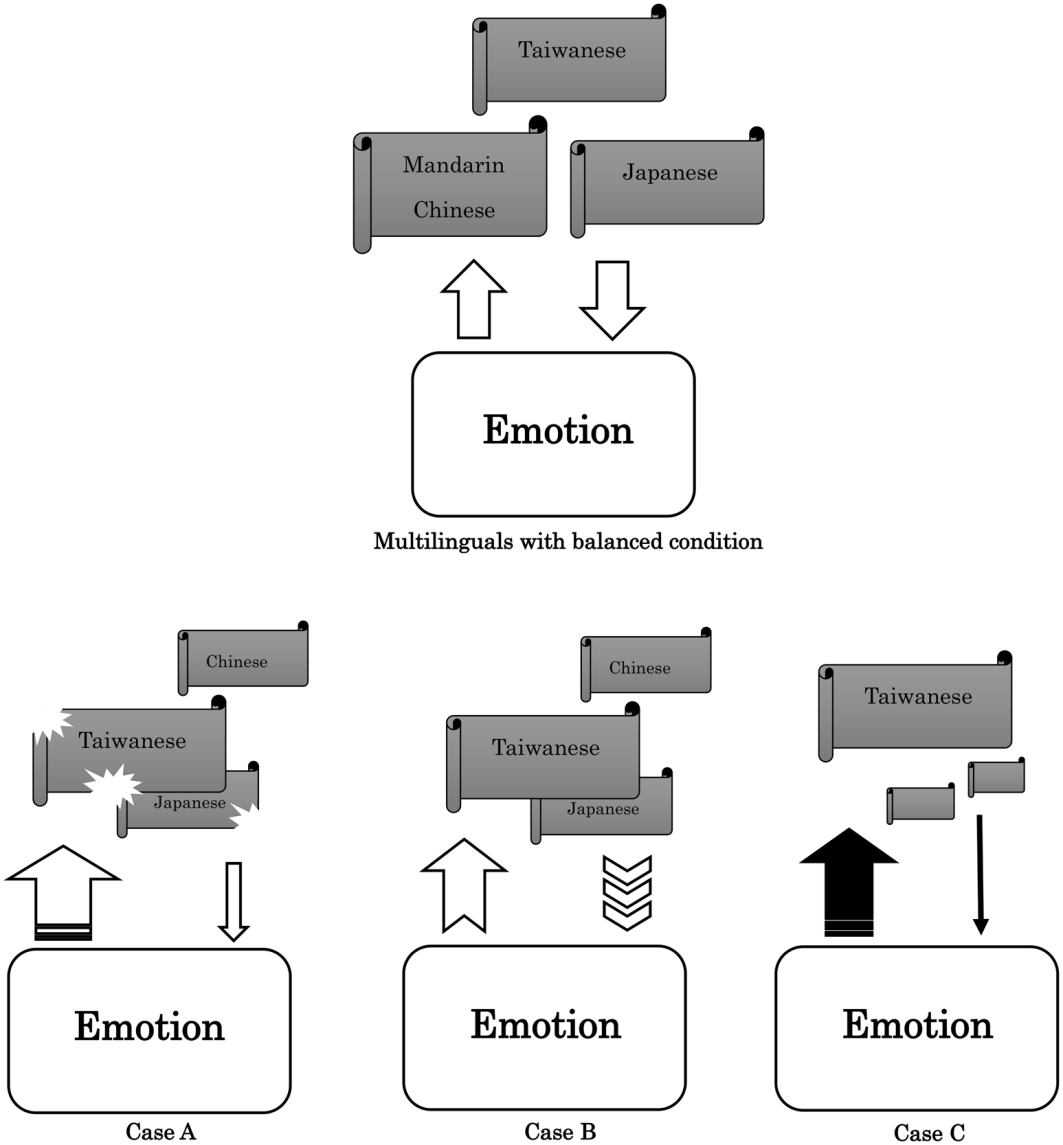
See the text.

According to the statement of Case A, their neighbor was a Japanese policeman and he always followed her. She told us that this Japanese policeman closely monitored her everyday life for almost one year. She did not understand why the government sent a Japanese policeman to watch her. She felt very angry about this incident and she claimed she wanted to sue the government. She would wait outside her house and shout bad words to the “Japanese policeman” if she met him. The truth was that her neighbor was truly Japanese. However, he was a Japanese teacher and he never talked to her. When she was asked why she thought her neighbor was following her, we found that she misunderstand his greeting of the Japanese style “you good” to the Taiwanese “die good”. After her families explained to the old lady, she continued to insist that her neighbor was a “Japanese policeman” who hoped for her to die. Under the insistence of the old lady, the whole family moved to another location. Her delusion also gradually disappeared after they moved. Her son told us that her mother had a very bad memory about Japanese policeman from her childhood, which could be the reason for her dramatic emotional response (see Table 3). In Case A, biased language information induced an over-reaction or inappropriate emotional response (see Fig 4). Compared with Case A-1 (with the same level of cognitive function and similar neuroimaging results), this kind of delusion could not be induced. For Case A-1, the same event would only be thought as due to the poor pronunciation of foreigners. Without the life experience and language background of Case A, no negative emotion or imagination would contribute to such common language mistakes.

**Table 3:** Language ability and related emotion triggered by language.

	Case A	Case B	Case C
Taiwanese	○	○	○
Japanese	Δ	▲	×
Mandarin Chinese	Δ	Δ	×
Emotion	+++	+	+++

**Note:** Three of our subjects (A, B, C) all speak fluent Taiwanese. Their Japanese and Mandarin Chinese level had degenerated to low level. In case A and C, strong emotion combined with language they perceived. The meaning of symbols is as following.

○: fluent

Δ: Low fluency, with only partially understanding and production

▲: Use Japanese only in counting numbers

×: Very low fluency

+~+++: Emotional response to languages besides mother language (Taiwanese).

### Case B

#### Summary of the clinical history and related language background

Age: 80

Chief complaint: Poor memory and persistent delusion

Past medical history: Idiopathic hypertension under regular control

Current diagnosis: Mild AD

Disease duration: 2 years

Previous Job before retirement: High school teacher

Language use in daily life: Mostly she communicated with people in Taiwanese. She talked with unfamiliar friends or school staffs in Mandarin Chinese. When she talked with her husband or when she did not want her children to understand, she used Japanese. (See Fig 3)

#### Detailed content of delusion in Case B

According to her son, her mother had delusion about her daughter in law stealing her money. When they had to deal with money with her, they needed to take photos. Her delusion was very stereotypical and was solely restricted to her daughter in law. When we asked her why her daughter in law was always involved, she told us her daughter in law was not good at numbers. The truth is that the old lady could only count numbers in Japanese. However, her daughter in law could only count numbers in Taiwanese or Mandarin Chinese. Subsequently, we found that after we asked her daughter in law to learn to count money in Japanese before her, the frequency and severity of delusion decreased surprisingly. Intermittently, she still insisted her money was stolen even with the photos and repeated counting. Under such circumstances, the family had to ask their father (her husband) to count again in Japanese, which would always relieve her delusion. For Case B-1, counting money in Japanese was just another way of counting money. Unlike Case B, a positive emotion or feeling of trust would be induced through Japanese speaking.

### Case C

#### Summary of clinical history and related language background

Age: 83

Chief complaint: Persistent delusion

Past medical history: Idiopathic hypertension, Type 2 DM, Congestive heart failure

Current diagnosis: Mild AD

Disease duration: 2 years

Previous Job before retirement: Street vendor in the market

Language use in daily life: Mostly she communicated with people in Taiwanese. She spoke Japanese or Mandarin Chinese only in the market and it depended upon the customers. After she retired, she almost exclusively used Taiwanese. (See Fig 3)

#### Detailed content of delusion in Case C

According to her son, her mother used to speak Taiwanese, Japanese and Mandarin Chinese very well before the onset of dementia. However, her language ability deteriorated thereafter. At present, she used Taiwanese (her mother tongue) most of the time. And she could not understand other people’s intermittently conversation. Her son was a businessman and he sometimes had to talk to other people on the phone. He found that her delusion would become aggravated after he talked to someone on the phone in Mandarin Chinese. Her delusion was so vivid that she once even called a policeman to report that she had been abandoned. In this case, they moved to the countryside where most people only speak Taiwanese in their everyday life. While her delusion persisted, it was not as frequent as in the city. Compared with Case C-1, unknown language is just another language. There was no connection with a past life memory or negative emotion. However, Mandarin Chinese and Japanese may have been related to a bad experience in the life of Case C.

## Discussion

### Summary of our results

In this study, we demonstrated that multilingual (T.J.C.) dementia patients exhibit more behavioral symptoms compared with their age- and education-matched bilingual (T.C.) controls from similar upbringings. More specifically, they have more delusional ideas. Multilingual patients also exhibited poor performance on the Boston naming test. Through detailed description of three of our cases, the relationship between the degenerated language ability and delusion is also presented.

### Limitation

In this study, there are several limitations of note. First of all, we did not fully examine language function by using more comprehensive language tools, because most of our subjects were very old and they would not likely be able to complete a neuropsychological test that took more than 30 minutes to complete. Second, our sample size was small, and more bilingual cases (control group) dropped out of our study, compared with the multilingual cases (Fig 1). This could increase the potential for bias on case selection. The small sample size is deficient of representativeness, and the small sample size would have effect on the statistical power of statistical analysis results. The reason the subjects dropped out of study was mostly due to personal health factors (they thought they were too old and too weak to undergo the neuropsychological tests). Another common reason the patients refused to participate this research is that they considered that their Japanese or Mandarin Chinese was too bad to be tested. In 38 subjects who dropped out our study, 18 of them were multilinguals and 20 of them are bilinguals. Also, we would better recruit monolingual AD patients as the controls; however, due to the specific situation of Taiwan, most people can use at least two languages.

### Language mixing, emotion and delusion

First of all, our patients were generally low proficiency multilinguals, which may partially explain why they were more vulnerable to Alzheimer’s disease. During the progression of AD, bilingual (or multilingual) patients tend to use inappropriate mixing of languages [19,20], which may inevitably lead to misunderstanding and confusion [21]. According to the language models proposed previously [22], highly-proficient bilinguals learned their language early and more comprehensively. Thus, they may use their second language more implicitly; in another words, they rely more on their procedural memory system. On the other hand, for the low-proficient bilinguals, their language ability relies more on declarative memory system. The declarative memory system is more vulnerable during the progression of Alzheimer’s disease. Therefore, asymmetric language degeneration will be expected more often in low-proficient compared with highly-proficient bilinguals. Consequently, more delusion or confusion may be triggered. In our cases, “theft” and “suspiciousness” were the most frequently observed behavioral symptoms. Both symptoms exhibit a very close relationship with misunderstanding.

Second, delusion can also be considered as a faulty attempt to balance cognition and emotion. Such attempts may represent the mediation of the conflict between “true” reality and “self-serving” reality [14]. Some reports even suggest that patients with delusion may have deficits in monitoring reality [23]. Confabulation, another common symptom in dementia patients, may also illustrate such attempts. In previous studies, confabulation had been proven to be associated with delusion and frontal lobe dysfunction [24,25]. In our patients, asymmetric degeneration in different languages may result in an imbalance between emotion and cognition (see Fig 4). In their condition, people speak more than one language due to their unique environment and history. They shared similar tragic experiences in their early life. As a result, they usually have more complex conflicts and emotional responses to language. In previous reports, delusion had been related to early life experiences or psychological trauma [26,27]. We would better recruit multilingual AD patients without similar early tragic experiences in order to control the influence of early life tragic experiences. However, this kind of “non-desktop” practical work prevented us from setting such sophisticated control. However, this is the first report to examine language background and delusion.

### Advanced degeneration among bilingual/multilingual AD patients

According to the cognitive reserve hypothesis, bilingual (or multilingual) people may compensate for the dementia pathology better and delay the onset of dementia [28]. After bilingual people begin to exhibit symptoms of dementia, they may deteriorate more rapidly than their monolingual peers. Furthermore, the greater number of behavioral symptoms that were observed might just be another sign of much more widespread brain degeneration. Early studies demonstrated that people with more cognitive reserve might exhibit more atrophic brain after dementia [29]. In this study, we also evaluated the medial temporal lobe in both groups using a visual rating scale (see Fig 2). However, we did not find any difference between the two groups. Perhaps a longer period of time is necessary to observe this possible mechanism.

Future investigations will hopefully clarify the effects of acetylcholinesterase inhibitors on our subjects. To date, it has proven to exhibit benefits on language impairment in AD [30] and to increase their life expectancy [31]. By contrast, we may need more specific psychosocial care for our subjects. It has been demonstrated that a familiar environment may be related to fewer incidences of wandering behaviors [32]. Similarly, familiar language could also further reduce the behavioral symptoms in multilingual patients. As noted in a previous study, the role of bilingual/bicultural caregiver will likely be increasingly significant in the future [33]. This also reminds of us the importance of person-centered care for dementia patients. Eventually, unique personal experiences and history may interact with the degenerated brain to shape the symptoms of patients.

## Supporting Information

S1 File Detailed neuropsychological tests results of participants in our study are revealed here. (XLSX)
